# Hypoxia evokes increased PDI and PDIA6 expression in the infarcted myocardium of ex-germ-free and conventionally raised mice

**DOI:** 10.1242/bio.038851

**Published:** 2018-11-30

**Authors:** Klytaimnistra Kiouptsi, Stefanie Finger, Venkata S. Garlapati, Maike Knorr, Moritz Brandt, Ulrich Walter, Philip Wenzel, Christoph Reinhardt

**Affiliations:** 1 Center for Thrombosis and Hemostasis (CTH), University Medical Center Mainz, Johannes Gutenberg University Mainz, Langenbeckstrasse 1, 55131 Mainz, Germany; 2 Center for Cardiology, Cardiology I, University Medical Center Mainz, 55131 Mainz, Germany; 3 German Center for Cardiovascular Research (DZHK), University Medical Center Mainz, Partner Site RheinMain, 55131 Mainz, Germany

**Keywords:** HL-1 cardiomyocytes, Hypoxia, Left anterior descending artery ligation, PDI, PDIA6, Germ-free

## Abstract

The prototypic protein disulfide isomerase (PDI), encoded by the *P4HB* gene, has been described as a survival factor in ischemic cardiomyopathy. However, the role of protein disulfide isomerase associated 6 (PDIA6) under hypoxic conditions in the myocardium remains enigmatic, and it is unknown whether the gut microbiota influences the expression of PDI and PDIA6 under conditions of acute myocardial infarction. Here, we revealed that, in addition to the prototypic PDI, the PDI family member PDIA6, a regulator of the unfolded protein response, is upregulated in the mouse cardiomyocyte cell line HL-1 when cultured under hypoxia. *In vivo*, in the left anterior descending artery (LAD) ligation mouse model of acute myocardial infarction, similar to PDI, PDIA6 protein expression was enhanced in the infarcted area (LAD+) relative to uninfarcted sham tissue or the neighbouring area at risk (LAD–) of C57BL/6J mice. Interestingly, we found that ex-germ-free (ex-GF) mice subjected to the LAD ligation model for 24 h had a reduced ejection fraction compared with their conventionally raised (CONV-R) SPF controls. Furthermore, the LAD+ area in the infarcted heart of ex-GF mice showed reduced PDIA6 expression relative to CONV-R controls, suggesting that the presence of a gut microbiota enhanced LAD ligation-triggered PDIA6 expression. Collectively, our results demonstrate that PDIA6 is upregulated in cardiomyocytes as a consequence of hypoxia. In the LAD mouse model, PDIA6 was also increased in the infarcted area under *in vivo* conditions, but this increase was suppressed in ex-GF mice relative to CONV-R controls.

This article has an associated First Person interview with the first author of the paper.

## Abstract (precis)

**Summary:** We identified PDIA6 as a hypoxia-induced element of the unfolded protein response in cardiomyocytes and infarcted mouse hearts. PDIA6 expression and ejection fractions were reduced in infarcted ex-germ-free mouse hearts.

## INTRODUCTION

Acute myocardial infarction (AMI) triggers the unfolded protein response (UPR) in cardiomyocytes to protect the ischemic surrounding from hypoxic stress (Thuerauf et al., 2006; Wang et al., 2018). The prototypic protein disulphide isomerase (PDI; encoded by *P4HB*), the prototypic PDI family member ensuring proper protein folding in the endoplasmic reticulum (ER), was previously shown to protect from myocardial infarction (Toldo et al., 2011a). However, there is an increase in P4HB levels with a paradoxical decrease of its active form in the infarcted diabetic mouse heart (Toldo et al., 2011b). PDI and other PDI family members are instrumental to ensure correct protein folding and to enhance superoxide dismutase 1 activity (Toldo et al., 2011a). Adenoviral transfection and overexpression of PDI in the mouse myocardium was shown to attenuate cardiac remodelling and to diminish cardiomyocyte apoptosis (Toldo et al., 2011a). Inside the vasculature, the oxidoreductase function of PDI is critical for the activation of cryptic blood-borne tissue factor on monocytes, the coagulation initiator that promotes arterial thrombus formation (Reinhardt et al., 2008). Furthermore, the oxidoreductase PDI regulates fibrin-mediated platelet aggregation (Lahav et al., 2002). PDI expression was also elevated in the myocardium of mice that were exposed to systemic hypoxia and in infarcted heart tissue (Tian et al., 2009). In addition to cardiomyocytes, the expression of PDI, which protects from apoptosis and promotes cell migration and adhesion, is also induced in endothelial cells (Tian et al., 2009). Hence, it is relevant to explore whether other PDI family members that are involved in the setting of AMI, are induced in cardiomyocytes.

In mammalian cells, three distinct pathways regulate the UPR: ER transmembrane inositol-requiring enzyme 1α (IRE1α), pancreatic ER kinase (PERK) and activating transcription factor 6 (ATF6). Hypoxia is a well-recognised activator of the UPR in cardiac myocytes (Thuerauf et al., 2006). The PDI family member PDIA6 (ERP5, TXNDC7) is an ER-resident protein that plays a crucial role in ER stress, as it limits the activation of the UPR by blocking the activity of the UPR sensor IRE1α through its direct binding to cysteine 148 and also acts on PERK (Eletto et al., 2014). An additional pool of PDIA6 acts as a cofactor of binding immunoglobulin protein (BiP; GRP-78), an ER resident chaperone (Jessop et al., 2009). Interestingly, the loss of PDIA6 does not result in ER stress and impaired protein folding (Rutkevich et al., 2010), but, vice versa, it has been demonstrated with neonatal rat ventricular myocytes that the PDIA6 underlies regulation by ATF6, which is activated by hypoxia, and that adenoviral overexpression of PDIA6 protects cardiomyocytes from stimulated ischemia/reperfusion-induced cell death (Vekich et al., 2012; Doroudgar et al., 2009).

Interestingly, recent research has revealed that impairment of the ER stress response in the gut epithelium is linked to intestinal dysbiosis and inflammation (Coleman et al., 2018; Kaser et al., 2008; Adolph et al., 2013), but it is currently unresolved whether the absence of a gut microbiota can impact the UPR in the intestine and whether hypoxia-regulated elements of the UPR are influenced by microbial colonisation at remote vascular sites, such as the myocardium. As an improved ER protein-folding capacity supports cardiomyocyte survival and PDIA6 plays a protective role by limiting the UPR, it is crucial to understand the conditions that determine hypoxia-induced PDIA6 expression in cardiomyocytes.

Because the influence of hypoxia on PDIA6, as it occurs during AMI, is unexplored, we here studied the impact of hypoxia (1% oxygen) on the expression of PDIA6 in HL-1 cardiomyocytes. To elucidate whether this UPR-regulator is influenced at the site of infarction *in vivo*, we analysed PDIA6 expression in a mouse model of AMI. Our results revealed that PDIA6 is upregulated in hypoxia-treated cardiomyocytes and in the infarcted cardiac tissue of mice subjected to left anterior descending artery (LAD) ligation. By analysing germ-free (GF) mice that were subjected to LAD ligation, we observed that the increase in PDIA6 expression in the murine myocardium was significantly reduced when the gut microbiota was absent, providing first evidence that the colonisation status of the host may impact the UPR in the infarcted myocardium.

## RESULTS

### Hypoxia increases the expression of PDIA6 in cultured HL-1 cardiomyocytes

**Fig. 1.**
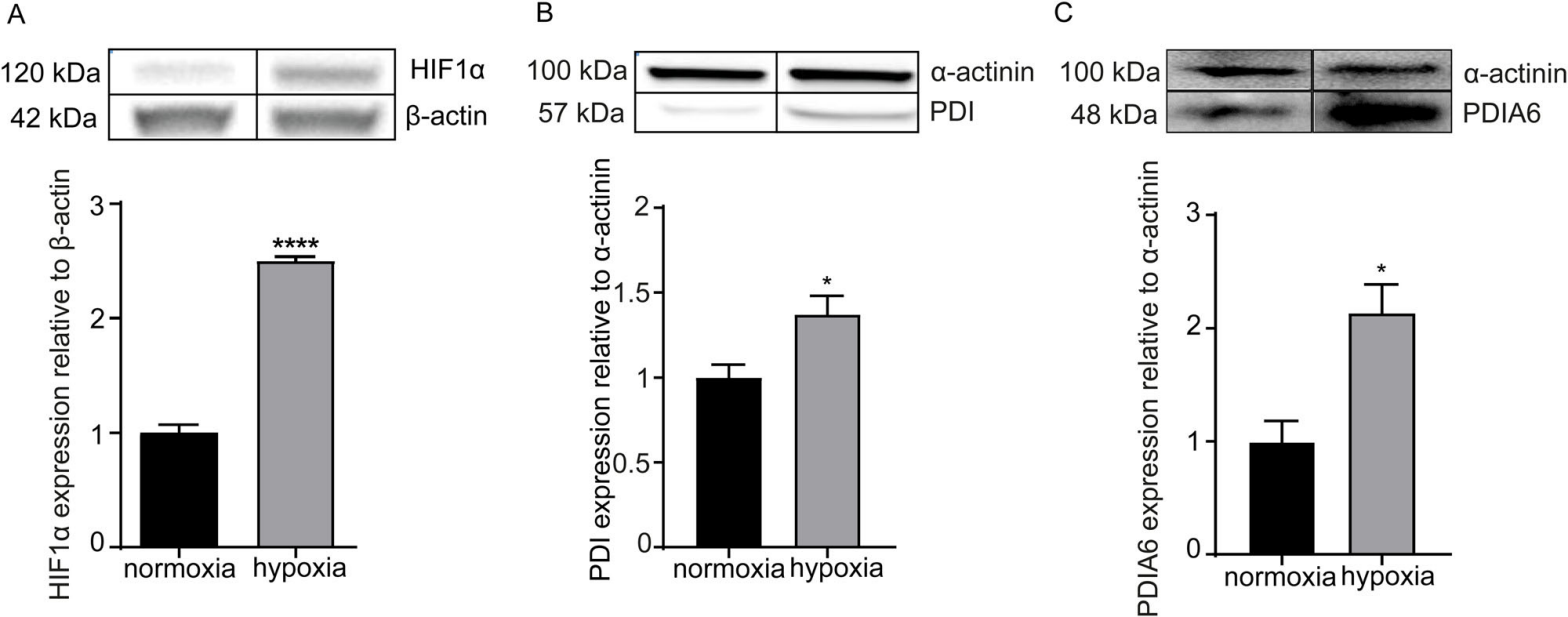
**Hypoxia-induced upregulation of HIF1α, PDI and PDIA6 in HL-1 cardiomyocytic cells.** (A) Hypoxia-dependent (1% O_2_) increase in HIF1α protein expression relative to β-actin (*n*=3, 24 h incubation). (B,C) Hypoxia-dependent (1% O_2_) increase in PDI (*n*=4, 24 h incubation) (B) and PDIA6 (*n*=3, 12 h incubation) (C) protein levels relative to α-actinin. Normoxia, black bars; hypoxia, grey bars. All data are expressed as mean±s.e.m. Statistical comparisons were performed using the Student's *t*-test; *P<0.05, ****P<0.0001.

Since PDIA6 has been suggested to be part of the ER stress response in cardiomyocytes, we used a hypoxic chamber to apply 1% hypoxia for 12 h and 24 h to cultured HL-1 cells (Claycomb et al., 1998). We confirmed the hypoxic conditions by the expected upregulation of hypoxia-inducible factor-1-α (HIF1α) after 24 h of incubation (Fig. 1A), which escapes degradation due to the absence of PHD-mediated hydroxylation under hypoxic conditions (Fig. S1) (Hölscher et al., 2011; Myllyharju, 2008). Strikingly, in addition to PDI (*P4HB*), our experiments comparing hypoxic HL-1 cardiomyocytes revealed that the protein levels of PDIA6 are likewise increased during hypoxia after 12 h (Fig. 1B,C). In contrast, at 24 h of incubation, only a tendency of increased PDIA6 expression was detected (data not shown). Of note, this increase in PDIA6 protein levels was not detected in human umbilical vein endothelial cells (HUVECs) (Fig. S2). Therefore, our results demonstrate that PDIA6, which was previously identified as a negative regulator of the UPR (Eletto et al., 2014), is increased by hypoxia in cultured cardiomyocytes.

### Expression of PDIA6 is enhanced in the infarcted mouse myocardium

**Fig. 2.**
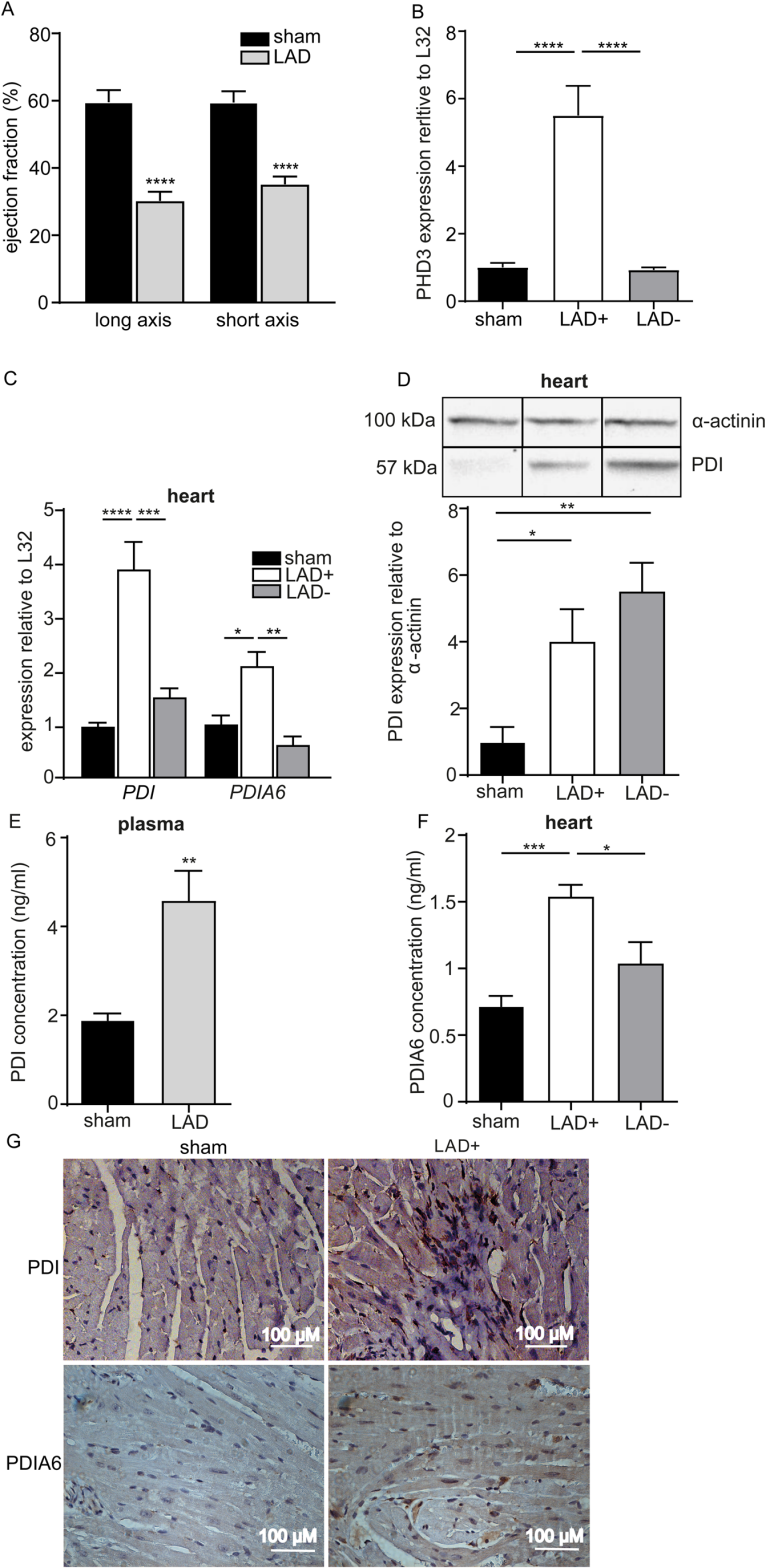
**The expression of PDI and PDIA6 is increased in the infarcted area (LAD+) in the 24 h LAD ligation mouse model.** (A) Long axis (*n*=6,9) and short axis (*n*=6,8) of the left ventricle after LAD ligation; SPF C57BL/6J mouse. (B) mRNA expression of the myocardial hypoxia marker PHD3 is increased relative to L32 in the LAD+ area compared with sham or LAD– tissues (*n*=4,5,5). (C) PDI and PDIA6 mRNA expression relative to L32 is increased in the LAD+ area compared with sham or LAD– tissues (*n*=5). (D,E) PDI protein levels are increased relative to α-actinin in the LAD+ (*n*=6) and LAD– (*n*=6) tissues compared to sham-operated heart tissue (*n*=5) (western blot) (D) and in plasma (*n*=6,8) (ELISA) (E). (F) PDIA6 protein levels are increased in LAD+ heart tissue relative to sham or LAD– tissues (ELISA) (*n*=5). (G) Immunohistochemistry for PDI (top row) and PDIA6 (bottom row) in sham-operated and infarcted LAD+ heart tissue (representative micrographs). Scale bars: 100 µm. LAD+ represents the ligated areas, LAD– represents the area proximal to ligation and sham represents the sham-operated animals. All data are expressed as mean±s.e.m. Statistical comparisons were performed using the Student's *t*-test and one-way ANOVA; *P<0.05, **P<0.01, ***P<0.001, ****P<0.0001.

We next interrogated whether PDIA6 protein levels are also upregulated in the infarcted tissue area, applying the LAD ligation mouse model of AMI. Infarction was confirmed by ultrasound imaging, indicated by a significant reduction of the left ventricular ejection fraction 24 h post-LAD ligation (Fig. 2A). This model causes hypoxic conditions in the infarcted area (LAD+), since hypoxia-regulated gene expression is elevated relative to sham tissue or the neighbouring area at risk (LAD−), as confirmed by elevated PHD3 expression levels (Fig. 2B) (Hirsilä et al., 2003; Rohrbach et al., 2005). Similar to the established role of PDI (*P4HB*) (Toldo et al., 2011a), the UPR regulator PDIA6 was increased in the infarcted LAD+ area relative to the LAD− area or tissues taken from the same site of sham-operated mice (Fig. 2C). In accordance, PDI showed enhanced protein levels in the LAD+ and LAD− area relative to the tissues of sham-operated mice (Fig. 2D). This increase was further reflected by increased plasma PDI levels (Fig. 2E). Interestingly, PDIA6 protein levels were threefold increased in the LAD+ area relative to specimens from sham-treated mice, and PDIA6 was significantly increased in the LAD+ area compared to the LAD− area (Fig. 2F). The increase of PDI and PDIA6 in the infarcted myocardium was further corroborated by immunohistochemistry of LAD+ specimens relative to sham-operated control tissue (Fig. 2G). In line with PDI, our results indicate that the hypoxia-dependent increase of PDIA6 found in HL-1 cardiomyocytes can be translated to the experimental conditions of AMI, where the arterial supply of oxygen is ceased in the infarcted LAD+ area.

### The gut microbiota determines the reduction in the ejection fraction and PDIA6 expression levels of the infarcted mouse heart

**Fig. 3.**
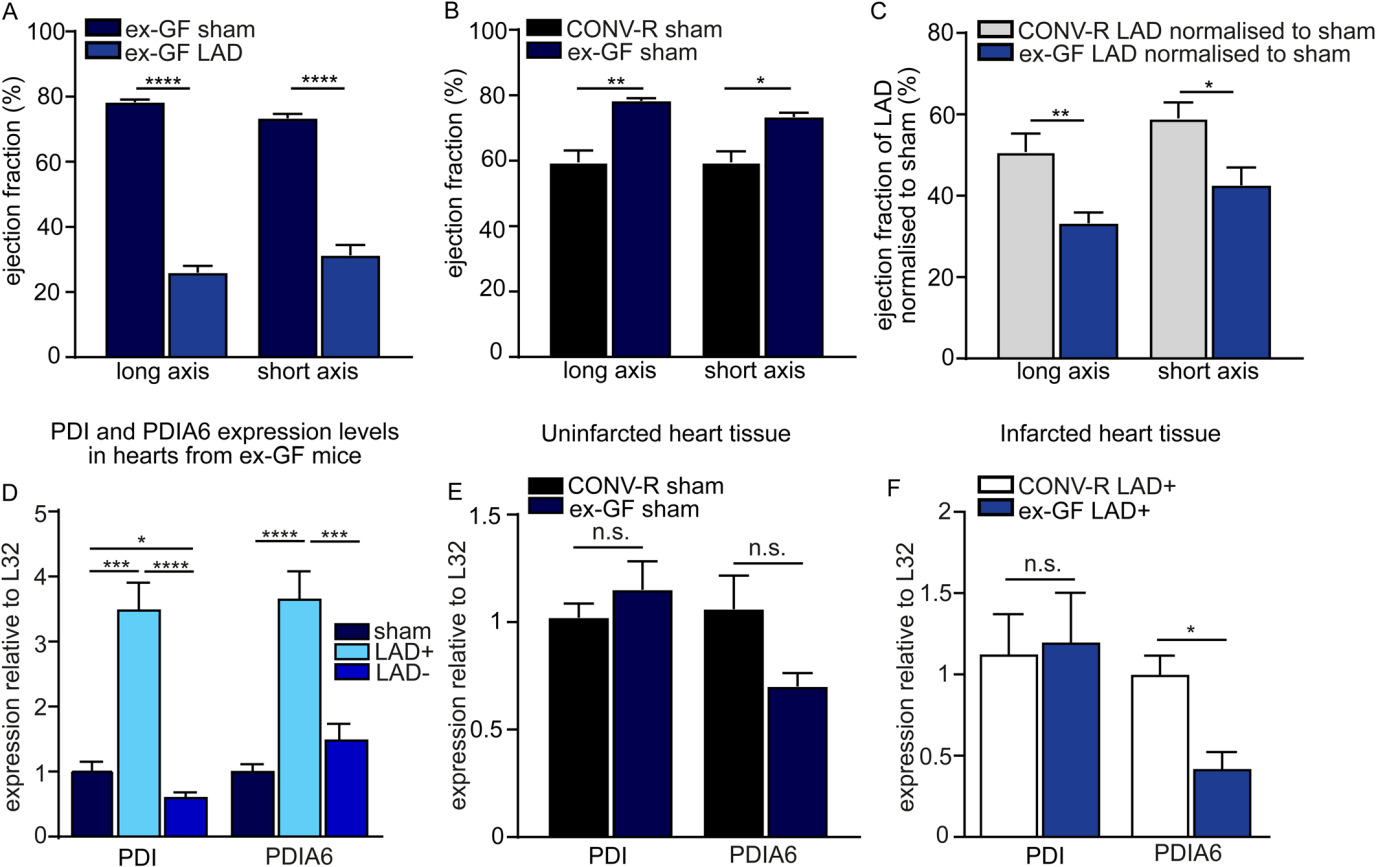
**24 h LAD ligation model of acute myocardial infarction in germ-free mice.** (A) Long axis (*n*=6,9) and short axis (*n*=6,8) of the left ventricle after LAD ligation of ex-germ-free (ex-GF) C57BL/6J mice as determined by ultrasound imaging. (B) Ejection fraction % of the long (*n*=6,3) and short axis (*n*=6,3) of sham-operated C57BL/6J mice kept under SPF conventionally raised (CONV-R) or ex-GF conditions. (C) Comparison of the long axis (*n*=9,9) and the short axis (*n*=8,8) of CONV-R SPF C57BL/6J mice, with ex-GF C57BL/6J mice normalised to sham-operated controls. (D) PDI (*n*=6,4,6) and PDIA6 (*n*=6,6,6) mRNA expression in sham-operated, LAD+ and LAD– heart tissues of ex-GF mice. (E) PDI and PDIA6 mRNA expression in heart tissues of sham-operated ex-GF mice compared to SPF CONV-R mice (*n*=6,3). (F) PDI (*n*=6) and PDIA6 (*n*=5) mRNA expression in LAD+ heart tissues of ex-GF mice compared to SPF CONVR mice. LAD+ represents the ligated area, LAD– represents the area proximal to ligation and sham represents the sham-operated animals. All data are expressed as mean±s.e.m. Statistical comparisons were performed using the Student's *t*-test or one-way ANOVA; n.s., not significant; *P<0.05, **P<0.01, ***P<0.001, ****P<0.0001.

As there is increasing evidence for the gut microbiota as a determinant of myocardial infarction (Lam et al., 2012), and because we found that the presence of a gut microbiota enhances angiotensin II-induced cardiac fibrosis (Karbach et al., 2016), we took advantage of the GF mouse model to explore whether the host colonisation status impacts on ventricular function and myocardial PDI expression under ischemic conditions in the LAD ligation model. Effective myocardial infarction was induced by LAD ligation in the GF mice, as demonstrated by ultrasound imaging of the left ventricle (Fig. 3A). Interestingly, the sham-operated ex-GF mice had a significantly reduced ejection fraction as compared to conventionally raised (CONV-R) controls (Fig. 3B). Intriguingly, we found a pronounced decrease of the ejection fraction in LAD-ligated ex-GF mice after we normalised to sham-operated ex-GF mice as compared to the normalised LAD-ligated CONV-R mice (Fig. 3C). As expected, also in the ex-GF mice, PDI and PDIA6 mRNA expression levels were increased in the LAD+ area relative to the LAD− area or sham-operated tissues of ex-GF mice that lack colonisation with a gut microbiota (Fig. 3D). In the left ventricle, PDI and PDIA6 transcript levels were unchanged under basal conditions, comparing sham-operated CONV-R and ex-GF mice (Fig. 3E). Most interestingly, when we compared the PDIA6 transcript levels in the LAD+ area of ex-GF mice with those of CONV-R mice, we noted significantly reduced PDIA6 expression in response to LAD ligation (Fig. 3F). This is in contrast to the transcript levels of PDI, which were unchanged in the LAD+ area between ex-GF and CONV-R mice (Fig. 3F). Hence, our results imply that the presence of a gut microbiota, which acts as a chronic inflammatory stimulus, can affect the LAD-ligation-induced UPR in the mouse myocardium.

## DISCUSSION

Our results show a direct upregulation of PDIA6 in cardiomyocytes due to hypoxic culture conditions. The *in vivo* relevance of increased PDIA6 in cardiomyocytes, an established regulator of the UPR, was further demonstrated under conditions of AMI in the murine model of myocardial infarction (permanent LAD ligation). Intriguingly, we found that the LAD-ligation-induced upregulation of PDIA6 was significantly reduced in the LAD+ area of ex-GF mice relative to their conventionally raised counterparts, providing first evidence that the UPR of the infarcted heart may be influenced by the colonisation status of the host.

In contrast to previous studies that identified PDIA6 in isolated rat cardiac myocytes, demonstrating the regulation via ATF6 and as an integral part of the ER stress response (Vekich et al., 2012; Eletto et al., 2014), we could, for the first time, demonstrate that hypoxia (1% oxygen) induced increased expression of this UPR-regulating factor in the HL-1 cardiomyocyte cell line. This finding is in line with the hypoxia-dependent upregulation of PDIA6 reported in a human cervix cancer cell line (SiHa) and a human head and neck cancer cell line (FaDu_DD_) (Sørensen et al., 2009). This indicates that the hypoxia-dependent upregulation of PDIA6 is not restricted to cardiac myocytes. Remarkably, because hypoxia-induced ATF6 upregulation protects rats from ischemia/reperfusion-induced necrosis (Jia et al., 2016), and PDIA6 is induced by ATF6 in ischemia/reperfusion and has a protective role in cardiomyocytes (Vekich et al., 2012), our results with HL-1 cells cultured at 1% oxygen atmosphere in a hypoxic chamber imply that PDIA6 mediates part of this protective hypoxia-dependent effect.

Hypoxia evokes the UPR in the ER, as shown in ventricular myocyte cultures (Thuerauf et al., 2006). To explore whether PDIA6 is a relevant hypoxia-regulated factor during AMI, we have tested the *in vivo* relevance of the identified hypoxia-induced PDIA6 upregulation, which is specific to cardiomyocytes in the mouse model of myocardial infarction by permanent LAD ligation, showing a vast increase in PDIA6 protein expression in the infarcted LAD+ area, but not in the LAD− area (area at risk). This is in contrast to prototypic PDI, which was also increased in the LAD− area. In a seminal study with adenoviral constructs that either increased or suppressed PDIA6 expression, it has been convincingly demonstrated that PDIA6 protects neonatal rat ventricular myocytes from stimulated ischemia/reperfusion-induced cell death (Vekich et al., 2012). Furthermore, previous molecular biological studies have revealed that hypoxia-dependent ATF6 promotes PDIA6 expression (Vekich et al., 2012) and that PDIA6 controls the attenuation of IRE1 and PERK signals, but does not impact on ATF6 signalling (Eletto et al., 2014). Thus, our result on the upregulation of PDIA6 in infarcted heart tissue is coherent with the established role of PDIA6 in attenuating the hypoxia-induced UPR that was identified in cell culture models.

The gut microbial ecosystem influences vascular physiology and there is emerging evidence for the implication of the gut microbiota as a determinant of the extent of myocardial infarction (Reinhardt et al., 2012; Lam et al., 2012, 2016). In previous work, we have identified that the colonisation status of the host affects angiotensin II-triggered cardiac fibrosis and tissue infiltration with myeloid cells (Karbach et al., 2016). Therefore, we applied the LAD ligation model to ex-GF mice to study the extent of the functional impairment of ventricular function in gnotobiotic mice, and to test whether the expression of UPR regulator PDIA6 is influenced by the absence of the gut microbiota. With ex-GF mice, we revealed that, in the absence of the gut microbiota, the LAD-ligation-induced increase in PDIA6, which suppresses the UPR in the ER, is severely perturbed. This was associated with an increased drop of the ejection fraction in ex-GF mice relative to CONV-R controls at 24 h of LAD-ligation-induced myocardial infarction. Of note, there is still no remodelling at this early time point. As the upregulation of PDIA6 protects neonatal rat ventricular cardiomyocytes from simulated ischemia/reoxygenation-induced cell death (Vekich et al., 2012), it is conceivable that the impaired cardiovascular function following LAD ligation in the ex-GF mouse model relative to CONV-R controls is due to the reduced upregulation of PDIA6 as part of the myocardial UPR.

Additional work with gnotobiotic mouse models is required to define the role of colonizing microbial communities in myeloid cell infiltration and cardiac remodelling during AMI. As the loss of PDIA6 does not result in significant ER stress and impaired protein folding (Rutkevich et al., 2010), it will be important to target PDIA6 in an *in vivo* mouse model of AMI to explore how this hypoxia-induced UPR regulator affects infarct size. Future studies should also delineate the exact pathways of the ER stress response that are regulated by the deficiency or increased activity of PDIA6 in AMI.

## MATERIALS AND METHODS

### Animals

C57BL/6J mice were 8- to 14-week-old male mice housed in the Translational Animal Research Center (TARC) of the University Medical Center Mainz under specific-pathogen-free (SPF) or GF conditions in EU type II cages, with two to five cage companions, standard autoclaved laboratory diet and water *ad libitum*, 22±2°C room temperature and a 12 h light/dark cycle. GF mice were maintained as a GF mouse colony in sterile flexible film mouse isolator systems. The GF status of mice was tested weekly by polymerase chain reaction (PCR) for detection of 16S rDNA and by bacterial culture. GF mice, named as ex-GF, were kept under standard SPF conditions following LAD ligation for 24 h. All groups of mice were age and weight matched and were free of clinical symptoms. All procedures performed on mice were approved by the local committee on legislation on protection of animals (Landesuntersuchungsamt Rheinland-Pfalz, Koblenz, Germany; G10-1-051).

### Cell culture

The HL-1 cell line, derivatives from the AT-1 mouse atrial cardiomyocyte tumour lineage (Claycomb et al., 1998), was maintained in Claycomb medium (Sigma-Aldrich, St. Louis, MI, USA) supplemented with 0.1 mM Norepineprin, 2 mM L-glutamine, 100 U/ml penicillin/streptomycin and 10% v/v fetal bovine serum. Cells were seeded in six-well plates, coated overnight with 0.02% w/v gelatin and 0.5% v/v fibronectin. Upon 80% confluency or more, cells were placed in the hypoxic chamber adjusted at 1% O_2_ and 5% CO_2_, at 37°C (Coy Laboratory Products, Grass Lake, MI, USA), washed and supplemented with hypoxic Claycomb medium, and then incubated for 12 h or 24 h in hypoxia or normoxia. After the desired time point, cells were washed and lysed for RNA or protein extraction.

### LAD ligation

Male mice (8 to 12 weeks old) underwent either permanent LAD ligation injury or sham operation. Mice were anaesthetised through intraperitoneal injection of midazolam [5 mg/kg body weight (BW)], medetomidine (0.5 mg/kg BW) and fentanyl (0.05 mg/kg BW). During surgery, mice were mechanically ventilated using a rodent ventilator. Ischaemia injury was induced by permanent occlusion of the LAD artery with an 8-0 prelene suture (Johnson & Johnson Medical, Norderstedt, Germany). The ischaemic area below ligation was reassured by visual examination of a bright colour in the occluded myocardium. Atipamezol (0.05 mg/kg BW) and flumazenil (0.01 ml/kg BW) were administered to the mice for analgesia after operation. Sham mice underwent the same operation procedure except coronary artery ligation. Mice were sacrificed 24 h after ligation by exsanguination in isoflurane anaesthesia.

### Ultrasound imaging

Mice were anaesthetised with isoflurane, and heart rate and temperature were monitored during examination. High-frequency ultrasound was performed by a Vevo 770 system (FUJIFILM, VisualSonics, Toronto, Canada) represented as ejection fraction (%). Images were obtained from the left parasternal long axis and short axis. In two-dimensional guided m-mode (2D), various parameters including left ventricular end-diastolic diameter (LVEDD) and left ventricular end-systolic diameter (LVESD) were measured in accordance with the American Society of Echocardiography.

### Preparation of protein extracts from cells or tissues

Samples were mechanically lysed in cell lysis buffer (50 mM Tris-HCl, 150 mM NaCl, 5 mM EDTA, 1% Triton X-100, pH 8) containing complete protease inhibitor cocktail tablets (Roche, Penzberg, Germany), passing through a 30G×1/2″ needle at least five times. The homogenates were incubated for 30 min on ice and centrifuged three times at 9000 ***g*** for 10 min at 4°C to remove insoluble debris. Tissues were homogenised in cell lysis buffer by the Tissue Lyser II (Qiagen, Hilden, Germany) for 5 min at 30 Hz. They were then kept on ice for 30 min and centrifuged three times at 9000 ***g*** for 10 min at 4°C to remove insoluble debris. Protein concentrations for cells and tissues were quantified using a DC Protein Assay Kit 2 (Bio-Rad, Hercules, CA, USA) according to the manufacturer's instructions.

### Western blot

Protein lysates were supplemented with 3× sample loading buffer [62.5mM Tris-HCl pH 6.8, 2.5% w/v sodium dodecyl sulfate (SDS), 0.002% w/v Bromophenol Blue, 5% v/v β-mercaptoethanol, 10% v/v glycerol] and the proteins were denaturated for 10 min at 99°C. Thirty microliters of the denaturated proteins were subjected to 8% SDS-polyacrylamide gel electrophoresis and were then transferred onto a nitrocellulose membrane. Unspecific binding was blocked with 5% bovine serum albumin in Tris-buffered saline (TBS) supplemented with Tween 20 (20 mM Tris-base, 137 mM NaCl, 0.05% v/v Tween 20) for 30 min, and the primary antibodies anti-HIF-1-alpha, anti-PDI EPR9498, anti-PDIA6 EPR11132 (Abcam, Cambridge, UK), anti-β-actin and anti-α-actinin (Cell Signaling Technology, Danvers, MA, USA) were incubated overnight at 4°C and 1:1000 dilution, with gentle agitation. The membrane was washed for 1 h with TBST buffer (150 mM NaCl, 7.7 mM Tris-HCl pH 7.5, 1% Tween 20) and the secondary antibody peroxidase anti-rabbit IgG (H+L) at 1:10.000 dilution (Vector Laboratories, Burlingame, CA, USA) was incubated for 1 h. The membrane was washed for 1 h with TBST buffer and incubated in luminol chemiluminescent substrate (Cell Signaling Technology) for 1 min. The membranes were developed by a Chemi Doc Touch imaging system (Bio-Rad) and the densitometry analysis was performed by using ImageJ software (https://imagej.nih.gov/ij/).

### Quantitative real-time PCR

Total RNA was isolated from the cells with the RNeasy Kit and from the tissues with the RNeasy Fibrous Tissue Mini Kit (Qiagen) according to the manufacturer's instructions. Total RNA (2 μg) was reverse transcribed with a High Capacity cDNA Reverse Transcription Kit (Applied Biosystems, Foster City, CA, USA), and SYBR green-based quantitative real-time PCR was performed with iQ SYBR Green Supermix (Bio-Rad). The oligonucleotide sequences used are listed in Table S1.

### Mouse PDI (*P4HB*) and PDIA6 ELISA

Enzyme-linked immunosorbent assays (ELISAs) for both PDI and PDIA6 were performed according to the manufacturer's instructions (MyBioSource.com, San Diego, CA, USA).

### Immunohistochemistry

Tissues were fixed in Roti^®^-Histofix 4% (Carl Roth, Karlsruhe, Germany) and provided to the Core Facility Histology of the University Medical Center Mainz for PDI with anti-P4Hb antibody (Abcam) and for PDIA6 (Aviva Systems Biology, San Diego, CA, USA) staining. All sections were analysed by light microscopy (Axia Lab.A1, Zeiss, Oberkochen, Germany).

### Statistical analysis

All values are expressed as mean±s.e.m. Data sets were analysed with Prism 6 (GraphPad Software, San Diego, CA, USA) using one-way analysis of variance (ANOVA) or unpaired Student's *t*-tests. Differences of *P*<0.05 were considered statistically significant.

## Supplementary Material

Supplementary information
