# Supplementary material for: Hypoxia evokes increased PDI and PDIA6 expression in the infarcted myocardium of ex-germ-free and conventionally raised mice
Source: Biol Open. 2018 Nov 30;8(1):bio038851. doi: 10.1242/bio.038851 (PMC6361221; doi:10.1242/bio.038851)
Supplement: Supplementary information [file biolopen-8-038851-s1.pdf]

Figure S1

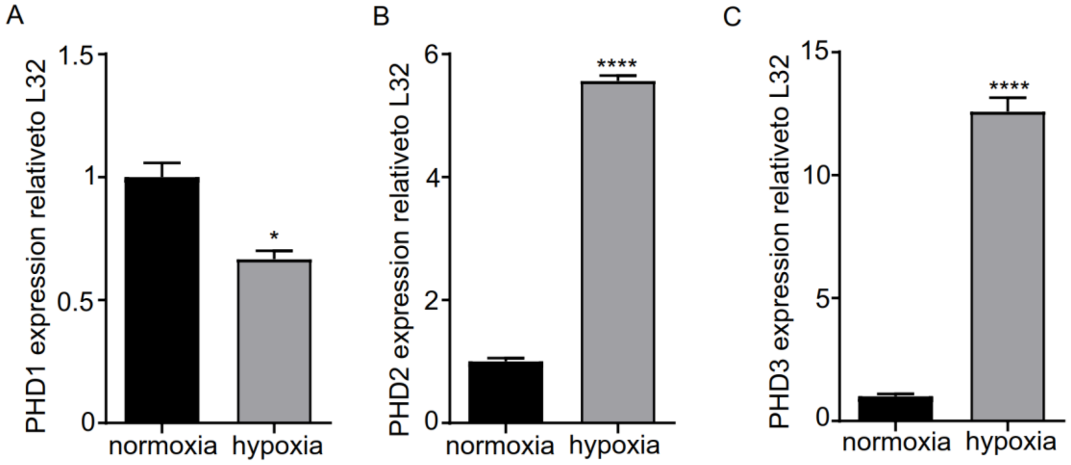

HIF Prolyl Hydroxylase expression of the HL-1 cells in hypoxia. (A) PHD1 (n=3), (B) PHD2 (n=3) and (C) PHD3 (n=3) expression relative to L32 of the HL-1 cells incubated for 24h in normoxia or hypoxia (1% O_2). All data were expressed as the means ± SEM. Statistical comparisons were performed using the Student's *t*-test * p<0.05, ****p<0.0001.

Biology Open • Supplementary information

Figure S2

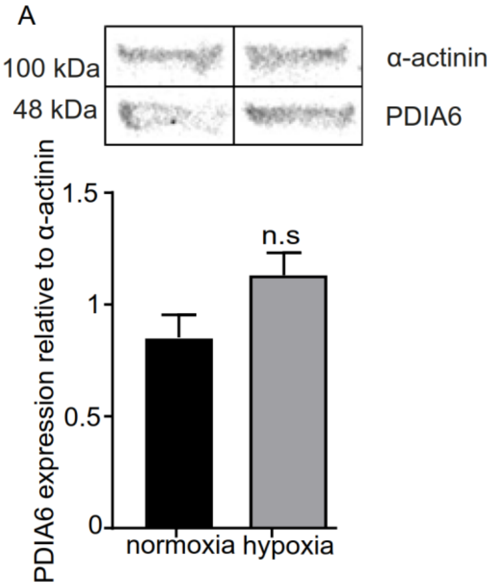

PDIA6 expression in HUVECs incubated in hypoxia. A. HUVECs incubated in normoxia or hypoxia (1% O_2) for 24h. Data were expressed as the means ± SEM. Statistical comparisons were performed using the Student's *t*-test.

Table S1. Oligonucleotide sequences used in quantitative real-time PCR.

Target	Gene		Sequence 5’ → 3′
Protein disulfide isomerase	P4HB	forward	CCGTGGCTACCCCACAATC
		reverse	GCAGTGTCAGACAGGGTTGTA
Protein disulfide isomerase family A member 6	PDIA6	forward	AGCTGCACCTTCTTTCTAGCA
		reverse	CAGGCCGTCACTCTGAATAAC
Hypoxia-Inducible Factor Prolyl Hydroxylase 2	EGLN1	forward	AGCTGGTCAGCCAGAAGAGT
		reverse	GCCCTCGATCCAGGTGATCT
Hypoxia-Inducible Factor Prolyl Hydroxylase 1	EGLN2	forward	AGTCCTTGGAGTCTAGCCGAAG
		reverse	TGGCAGTGGTCGTAGTAGCA
Hypoxia-Inducible Factor Prolyl Hydroxylase 3	EGLN3	forward	AGGCAARGGTGGCTTGCTATC
		reverse	GCGTCCCAATTCTTATTCAGGT
ribosomal protein L32	L32	forward	CCTCTGGTGAAGCCCAAGATC
		reverse	TCTGGGTTTCCGCCAGTTT
